# Prognosis Can Be Predicted More Accurately Using Pre- and Postchemoradiotherapy Carcinoembryonic Antigen Levels Compared to Only Prechemoradiotherapy Carcinoembryonic Antigen Level in Locally Advanced Rectal Cancer Patients Who Received Neoadjuvant Chemoradiotherapy

**DOI:** 10.1097/MD.0000000000002965

**Published:** 2016-03-11

**Authors:** SooYoon Sung, Seok Hyun Son, Chul Seung Kay, Yoon Suk Lee

**Affiliations:** From the Department of Radiation Oncology (SYS, SHS, CSK); and Department of Surgery (YSL), Incheon St. Mary's Hospital, College of Medicine, The Catholic University of Korea, Seoul, Korea.

## Abstract

We aimed to evaluate the prognostic value of a change in the carcinoembryonic antigen (CEA) level during neoadjuvant chemoradiotherapy (nCRT) in patients with locally advanced rectal cancer. A total of 110 patients with clinical T3/T4 or node-positive disease underwent nCRT and curative total mesorectal resection from February 2006 to December 2013. Serum CEA level was measured before nCRT, after nCRT, and then again after surgery. A cut-off value for CEA level to predict prognosis was determined using the maximally selected log-rank test. According to the test, patients were classified into 3 groups, based on their CEA levels (Group A: pre-CRT CEA ≤3.2; Group B: pre-CRT CEA level >3.2 and post-CRT CEA ≤2.8; and Group C: pre-CRT CEA >3.2 and post-CRT CEA >2.8). The median follow-up time was 31.1 months. The 3-year disease-free survival (DFS) rates of Group A and Group B were similar, while Group C showed a significantly lower 3-year DFS rate (82.5% vs. 89.5% vs. 55.1%, respectively, *P *= 0.001). Other clinicopathological factors that showed statistical significance on univariate analysis were pre-CRT CEA, post-CRT CEA, tumor distance from the anal verge, surgery type, downstage, pathologic N stage, margin status and perineural invasion. The CEA group (*P* = 0.001) and tumor distance from the anal verge (*P* = 0.044) were significant prognostic factors for DFS on multivariate analysis. Post-CRT CEA level may be a useful prognostic factor in patients whose prognosis cannot be predicted exactly by pre-CRT CEA levels alone in the neoadjuvant treatment era. Combined pre-CRT CEA and post-CRT CEA levels enable us to predict prognosis more accurately and determine treatment and follow-up policies. Further large-scale studies are necessary to validate the prognostic value of CEA levels.

## INTRODUCTION

In locally advanced rectal cancer, neoadjuvant chemoradiotherapy (nCRT) followed by curative surgical resection has been adopted as a standard treatment.^1–4^ nCRT improves local tumor control significantly, but the response to nCRT is not the same in all patients. Those with a good response to nCRT are likely to experience less recurrence.^5–7^ Patients with similar clinical features at the time of initial diagnosis could show different prognoses according to the response to nCRT.

Serum carcinoembryonic antigen (CEA) is a tumor marker used in colorectal cancer patients.^8^ Before the adoption of nCRT, the value of the initial serum CEA level at diagnosis was studied as a prognostic factor. An elevated initial serum CEA level has been known to be associated with poor prognosis. In the study reported by Wanebo et al,^9^ stage III colorectal cancer patients with preoperative CEA levels <5 ng/mL showed a longer median disease-free survival (DFS) time compared with those with CEA levels >5 ng/mL (28 months vs. 13 months). Wang et al^10^ also reported that a preoperative CEA level of >5 ng/mL was an independent prognostic factor on multivariate analysis.

As patients showed different prognoses according to the response to nCRT, several studies suggested that a change in serum CEA levels during nCRT would reflect the individual response and had a predictive value for prognosis.^11–15^ Patients who respond poorly to nCRT tend to have persistently elevated serum CEA levels after nCRT (post-CRT CEA). Not only initial CEA levels at diagnosis (pre-CRT CEA) but also post-CRT CEA levels might be considered prognostic factors. However, prognostic values of pre-CRT CEA and post-CRT CEA in the nCRT era and their meaningful cut-off values have not been established yet.

In this study, we aimed to evaluate the prognostic values of pre- and post-CRT CEA levels, and propose the optimal cut-off values to predict the DFS in locally advanced rectal cancer patients who underwent nCRT followed by curative surgical resection.

## METHODS

### Patients

Eligibility criteria were as follows: histologically proven adenocarcinoma in the rectum or, in a few cases, radiologically confirmed rectal cancer; clinical T3/T4 or node-positive disease on evaluation before nCRT; treated with nCRT followed by curative total mesorectal excision (TME); no evidence of distant metastasis at the time of diagnosis; CEA levels measured before nCRT, after nCRT, and after surgery; and Eastern Cooperative Oncology Group (ECOG) performance score 0–2.

Between February 2006 and December 2013, a total of 110 patients were identified to meet the above eligibility criteria for this study. All of the patients underwent nCRT and curative resection at Incheon St. Mary's Hospital. Among the 110 patients, 74 (67.3%) were men and 36 (32.7%) were women. The median age was 59 years (range, 27–84 years). The median follow-up time was 31.1 months. The patients’ clinical data were retrospectively collected following Institutional Review Board approval. Patients’ clinicopathologic characteristics are listed in Table 1.

**TABLE 1:**
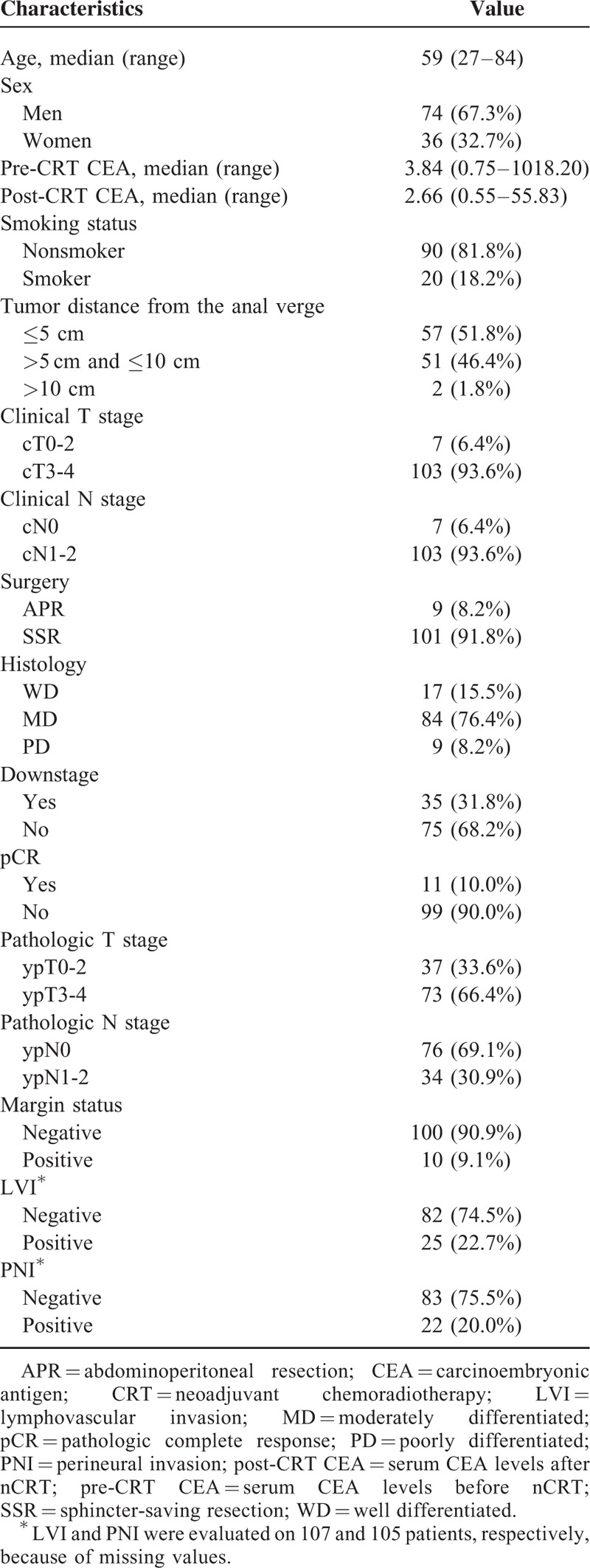
Clinicopathologic Characteristics

Before nCRT, all patients were evaluated with staging workups, including history and physical examination, complete blood count, liver function tests, CEA level, colonoscopy, chest X-ray, computed tomography (CT) scan of the abdomen/pelvis and chest, and pelvic magnetic resonance imaging (MRI). The stage of each patient was re-evaluated according to the seventh edition of the TNM classification of the American Joint Committee on Cancer (AJCC).^16^ CEA levels were measured before nCRT, between completion of nCRT and surgery, and again after surgery.

### Treatment

Radiotherapy (RT) was delivered to the whole pelvis at a dose of 45 Gy in 25 fractions, followed by a boost to the primary tumor of 5.4 Gy in 3 fractions, up to a total of 50.4 Gy in 28 fractions. Patients were treated in the prone position to reduce the volume of bowel within the pelvis. A conventional 3-field technique was used with posteroanterior, right lateral, and left lateral beams. The superior border was the L5/S1 junction and the lower border was 3 cm distal to the tumor or the lowest margin of the obturator foramen. The lateral border of the pelvis anterior field was 1.5 to 2 cm lateral to the widest bony margin of the true pelvic wall. For the lateral field, the anterior border was the posterior margin of the symphysis pubis and the posterior border was 1 cm behind the posterior margin of the sacrum. The boost field margin was 3 cm superior and inferior and 2 cm lateral to the primary tumor. The median RT duration was 38 days (range, 35–45 days).

Intravenous (IV) 5-fluorouracil- (5-FU) or oral capecitabine-based chemotherapy was administered concurrently with RT. 5-FU based chemotherapy was given to 103 patients (93.6%) at a dose of 425 mg/m^2^/day of 5-FU and 20 mg/m^2^/day of leucovorin during the first and fifth weeks of RT. Oral capecitabine at a dose of 1650 mg/m^2^/day was given to 7 patients (6.4%) daily during the whole period of RT.

Surgery was performed by an experienced colorectal surgeon at 6 to 8 weeks after completion of nCRT. All patients underwent radical surgical resection including TME, and if necessary, pelvic node dissection was performed. All surgical specimens were evaluated by experienced pathologists. The pathologic response to nCRT was evaluated using a tumor regression grade system proposed by Dworak et al.^17^ Circumferential radial margin (CRM) involvement was defined as involvement within 1 mm of the CRM. Histologic type, grade, pathologic staging, margin status, lymphovascular invasion (LVI), and perineural invasion (PNI) were evaluated on pathologic examination.

All patients, regardless of pathologic stage, were considered for adjuvant chemotherapy. 5-FU/leucovorin (LF) was given to 99 patients (90.0%). The chemotherapy regimen was changed to 5-FU/oxaliplatin/leucovorin (FOLFOX) or 5-FU/irinotecan/leucovorin (FOLFIRI) when a local recurrence or distant metastasis was detected during the course of chemotherapy. Two patients (1.8%) received adjuvant FOLFOX chemotherapy as a first-line regimen because of early recurrences detected right after surgery. Oral capecitabine and tegafur/uracil were each administered to 1 patient (0.9%), respectively. Seven patients (6.4%) did not receive adjuvant chemotherapy due to patient's refusal or poor performance status.

### Follow-Up

After the completion of treatment, the patients were regularly followed up with physical examinations, complete blood counts, liver function tests, chest radiography, and serum CEA levels every 3 months for the first 2 years. Pelvis and chest CT scans were performed every 6 to 12 months for at least 5 years. Patients also underwent follow-up colonoscopy within 1 year postoperatively and then once every 2 to 3 years. Patients in whom recurrence was suspected underwent specific examinations. Recurrences were diagnosed either pathologically or radiologically. Locoregional recurrence was defined as any recurrence in the pelvis, and distant metastasis as any recurrence outside the pelvis.

### Statistical Analyses

The *χ*^2^ test, Fisher exact test, independent *t* test, or one-way analysis of variance (ANOVA) were performed according to the nature of the variables. The maximally selected log-rank test in R version 3.1.2 (R Development Core Team, Vienna, Austria) was used to determine optimal CEA cut-off values.^18^ DFS, locoregional recurrence-free survival (LRFS), distant metastasis-free survival (DMFS), and overall survival (OS) were analyzed using the Kaplan–Meier method. DFS was defined as the time from the initial day of nCRT to recurrence or death or last follow-up. The last follow-up observation for DFS was censored if the patient was alive or lost to follow-up. LRFS and DMFS were defined as the time from the initial day of nCRT to locoregional recurrence and distant metastasis, respectively. OS was defined as the time from the initial day of nCRT to death. Univariate and multivariate analyses were performed using the Cox proportional hazards model. A *P* value of <0.05 was considered significant. Missing data were included in LVI and PNI. Only cases with available data on each variable were analyzed. LVI and PNI were evaluated on 107 and 105 patients, respectively.

## RESULTS

### CEA Grouping

Optimal cut-off values to predict the prognosis were calculated using the maximally selected log-rank test. Both CEA levels and DFS were used as the variables for the analyses. According to the results of the test, 3.2 and 2.8 ng/mL were adopted as cut-off values for pre-CRT CEA and post-CRT CEA levels, respectively.

Using the pre-CRT CEA and post-CRT CEA cut-off values, the 110 patients in this study were categorized into 3 CEA groups as per their CEA levels: Group A, pre-CRT CEA ≤3.2 ng/mL (n = 50); Group B, pre-CRT CEA >3.2 ng/mL and post-CRT CEA ≤2.8 ng/mL (n = 19); and Group C, pre-CRT CEA >3.2 ng/mL and post-CRT CEA >2.8 ng/mL (n = 41). The median pre-CRT CEA levels for Group A, Group B, and Group C were 1.95, 5.80, and 7.95 ng/mL, respectively, and the median post-CRT CEA levels were 1.70, 2.21, and 4.65 ng/mL, respectively. The clinicopathologic features of the 3 groups are shown in Table 2. Pre-CRT CEA, post-CRT CEA, downstage, pathologic complete remission (pCR), pathologic T stage (pT stage), and margin status showed significant differences between groups (*P* < 0.001, <0.001, 0.001, 0.036, <0.001, and 0.012, respectively). Patients in Group A showed a higher percentage of downstage, pCR, and pT stage 0-2 compared with Group B and Group C. The percentage of positive margins was higher in Group C. Group C patients showed a tendency toward a higher rate of pathologic positive nodes (*P* = 0.071), but this did not reach statistical significance. Age, gender, smoking status, tumor distance from the anal verge, clinical T stage, clinical N stage, surgery type, histology, presence of LVI, and presence of PNI were not different among the 3 groups.

**TABLE 2:**
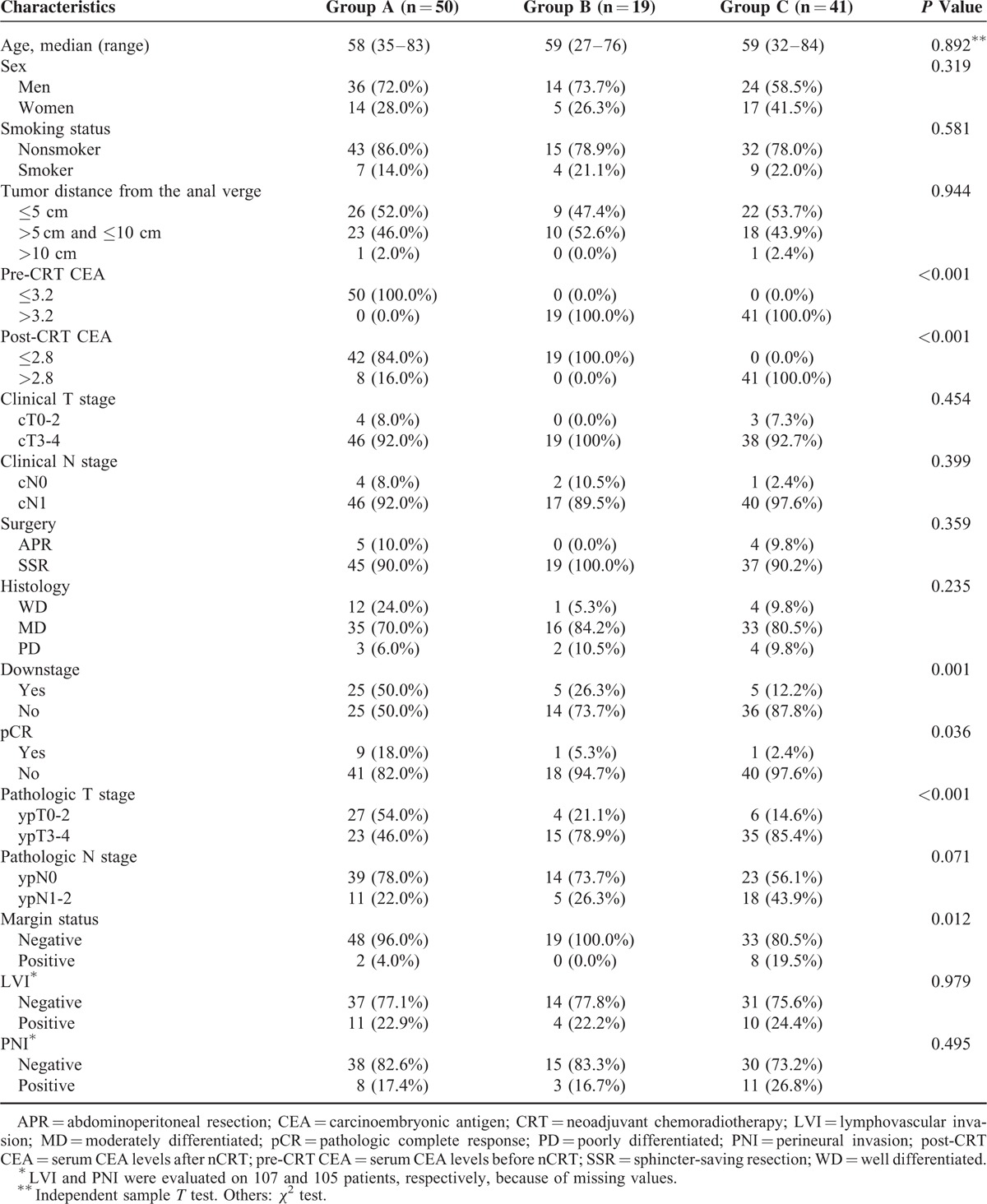
Patients Characteristics According to Carcinoembryonic Antigen Group

### Pattern of Failures

Of the 110 patients, locoregional recurrence occurred in 8 patients (7.3%) and distant metastasis was observed in 23 patients (20.9%). The common sites of distant metastasis were liver (12 patients) and lung (10 patients). In Group A, metastasis occurred in 4 patients, and the sites were liver, lung, and inguinal node in 1 patient each, respectively, while the fourth patient experienced peritoneal seeding, bone metastasis, and supraclavicular lymph node involvement. Only 1 patient in Group B showed metastasis, to the liver. The remaining 18 patients with metastasis were in Group C. For the patients in Group C, involvement of the liver was seen in 10 patients, lung in 9 patients, para-aortic node in 4 patients, inguinal node in 2 patients, and bone in 2 patients, while peritoneal seeding was observed in 1 patient.

### Disease-Free Survival

**FIGURE 1:**
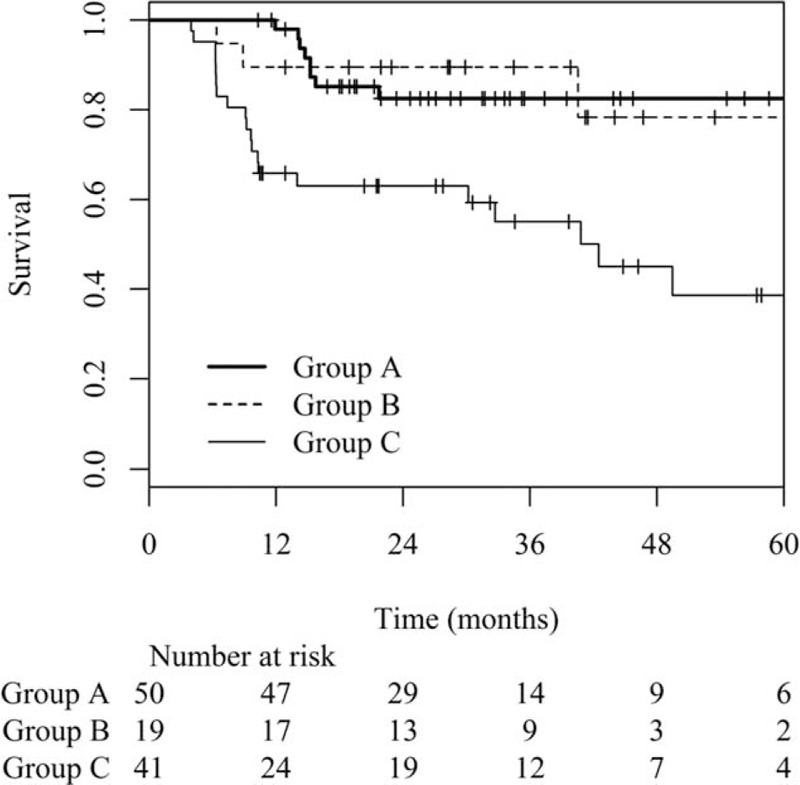
Disease-free survival curves based on the carcinoembryonic group (Group A: pre-CRT CEA ≤3.2 ng/mL, Group B: pre-CRT CEA >3.2 ng/mL and post-CRT CEA ≤2.8 ng/mL, Group C: pre-CRT CEA >3.2 ng/mL and post-CRT CEA >2.8 ng/mL).

For all patients, the 3-year DFS rate was 73.1%. The 3-year DFS rates for each group were as follows: Group A, 82.5%; Group B, 89.5%; Group C, 55.1%. The DFS rates were not different between Group A and Group B, but the rate was significantly lower in Group C (*P* = 0.001) (Figure 1). The 3-year LRFS rate for all patients was 82.5%. The 3-year LRFS rate of Group C showed a tendency to be lower than those of Group A and Group B, but the difference did not reach statistical significance (Group A, 86.4%; Group B, 94.7%; Group C, 71.8%, *P* = 0.061). The 3-year DMFS rate was 75.7%. The 3-year DMFS rate was significantly lower in Group C than in Group A and Group B (Group A, 89.4%; Group B, 89.5%; Group C, 54.0%, *P* < 0.001). The median DFS, LRFS, and DMFS were not reached at the median of 31.1 months of follow-up.

### Predictive Factors for Disease-Free Survival

We investigated possible clinicopathologic variables to determine the predictive factors for DFS. On univariate analysis, tumor distance from the anal verge, pre-CRT CEA, post-CRT CEA, surgery type, downstage, pathologic N stage, margin status, PNI, and CEA group were significantly associated with DFS (*P* = 0.016, 0.010, 0.004, 0.048, 0.031, 0.035, 0.035, 0.041, and 0.001, respectively; Table 3). Multivariate analysis was conducted using the significant factors from univariate analyses and revealed that CEA group and tumor distance from the anal verge were independent prognostic factors for DFS (*P* = 0.001 and 0.044, respectively).

**TABLE 3:**
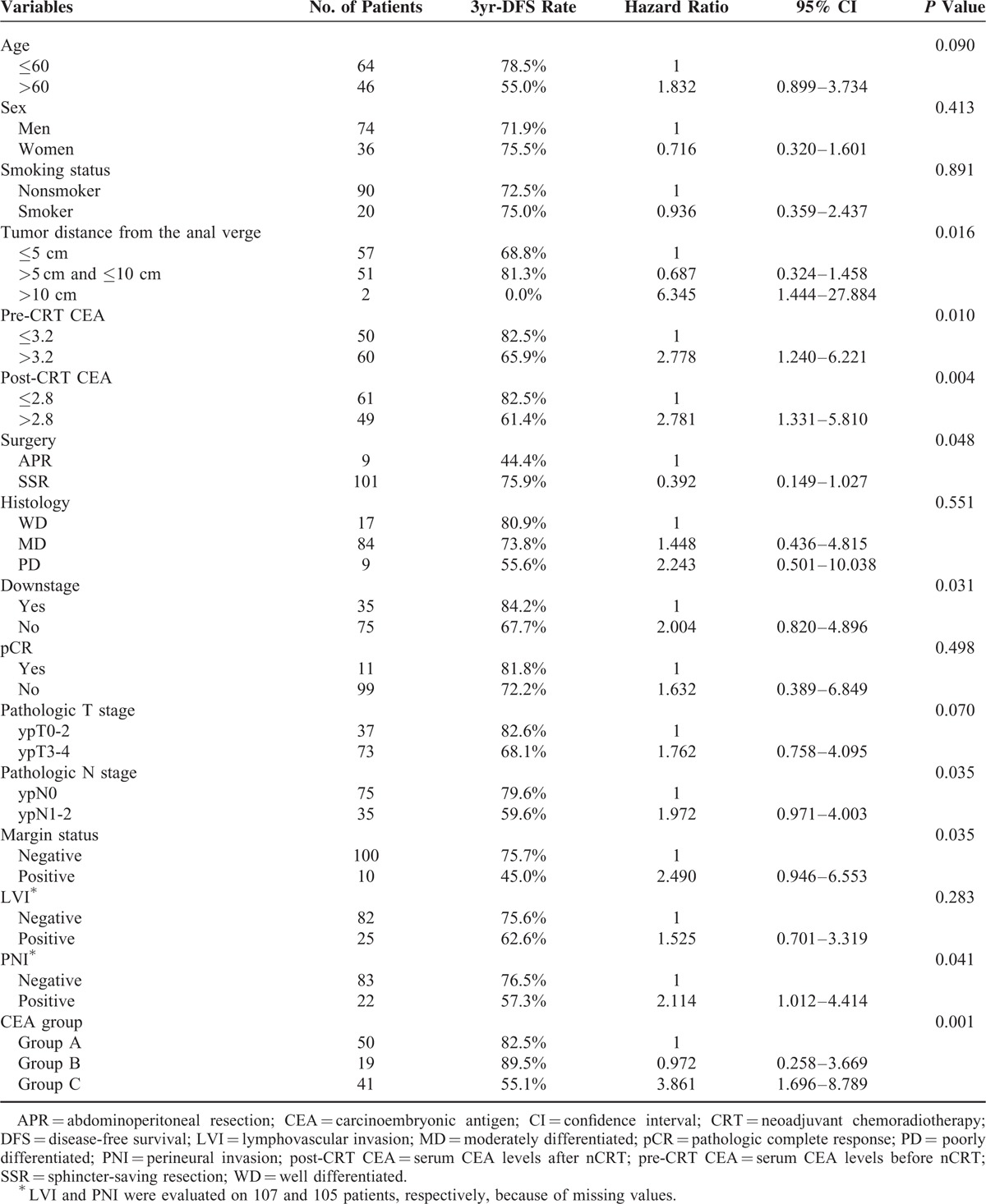
Univariate Analysis of Prognostic Factors

## DISCUSSION AND CONCLUSIONS

CEA is a well-established and most widely used tumor marker to predict prognosis and detect recurrence in patients with colorectal cancer.^8,19^ Increased CEA level has been found to be correlated with an advanced stage of disease.^20,21^ Wanebo et al^9^ reported that the recurrence rate was higher in patients who had a CEA level >5 ng/mL at diagnosis. Also, a rise in the serum CEA level after surgery was found to be an indicator of recurrence, preceding the clinical symptoms.^20^ According to Chu report, 89% of the patients who had intra-abdominal recurrences showed serum CEA levels >5 ng/mL.^22^

nCRT followed by curative surgical resection has become the gold standard treatment in locally advanced rectal cancer patients.^1–4^ After the adoption of nCRT, response to nCRT was found to influence the prognosis. Several reports suggested that the post-CRT CEA level could be used as a prognostic factor, reflecting the response to nCRT. Park et al^11^ reported that not only pre-CRT CEA but post-CRT CEA levels could be helpful to predict prognosis. They categorized patients into 3 groups according to pre/post-CRT CEA levels. Patients with pre- and post-CRT CEA levels >3 ng/mL displayed a significantly lower 3-year DFS than patients with pre-CRT CEA levels >3 ng/mL and post-CRT CEA levels ≤3 ng/mL. These results showed that the pre-CRT CEA level alone is not sufficiently effective to predict the prognosis in patients who received nCRT.

In the current study, we grouped the patients according to a pre-CRT CEA level of 3.2 ng/mL and a post-CRT CEA level of 2.8 ng/mL (Group A, pre-CRT CEA ≤3.2 ng/mL; Group B, pre-CRT CEA >3.2 ng/mL and post-CRT CEA ≤2.8 ng/mL; Group C, pre-CRT CEA >3.2 ng/mL and post-CRT CEA >2.8 ng/mL). Patients in Group A showed a better prognosis compared with patients in Group C who had elevated serum levels of both, pre- and post-CRT CEA. Despite a high pre-CRT CEA level, the 3-year DFS in patients of Group B was not significantly different than that in patients of Group A (Group A, 82.5%; Group B, 89.5%; Group C, 55.1%). A decrease in CEA level as a response to nCRT is associated with an increased DFS rate in our study. On multivariate analysis, CEA group was the independent prognostic factor associated with DFS. Jang et al^12^ reported that patients with a high pre-CRT CEA level and decreased post-CRT CEA level showed no difference in 3-year DFS compared with patients with a nonelevated pre-CRT CEA level. The 3-year DFS rate was significantly lower in patients with a pre-CRT CEA level >3.5 ng/mL and a post-CRT CEA level >2.7 ng/mL compared with the other 2 groups (52.6% vs. 94.7% vs. 88.0%, respectively, *P* < 0.001). Another study by Kim et al^14^ grouped patients according to pre-CRT CEA level and degree of reduction from pre-CRT CEA level to post-CRT CEA level (Group A, pre-CRT CEA ≤6 ng/mL; Group B, pre-CRT CEA >6 ng/mL and post-CRT CEA reduced ≥70% compared with pre-CRT CEA; Group C, pre-CRT CEA >6 ng/mL and post-CRT CEA reduced <70% compared with pre-CRT CEA). The 5-year DFS rate was similar in the first and second groups but significantly lower in the last group (76.0% vs. 66.0% vs. 39.5%, respectively, *P* < 0.001). Other reports analyzing the prognostic value of CEA group are listed in Table 4. Reviewing the reports, patients with decreased post-CRT CEA levels did not show inferior prognosis despite elevated pre-CRT CEA levels, compared with the patients with nonelevated pre-CRT CEA levels.

**TABLE 4:**
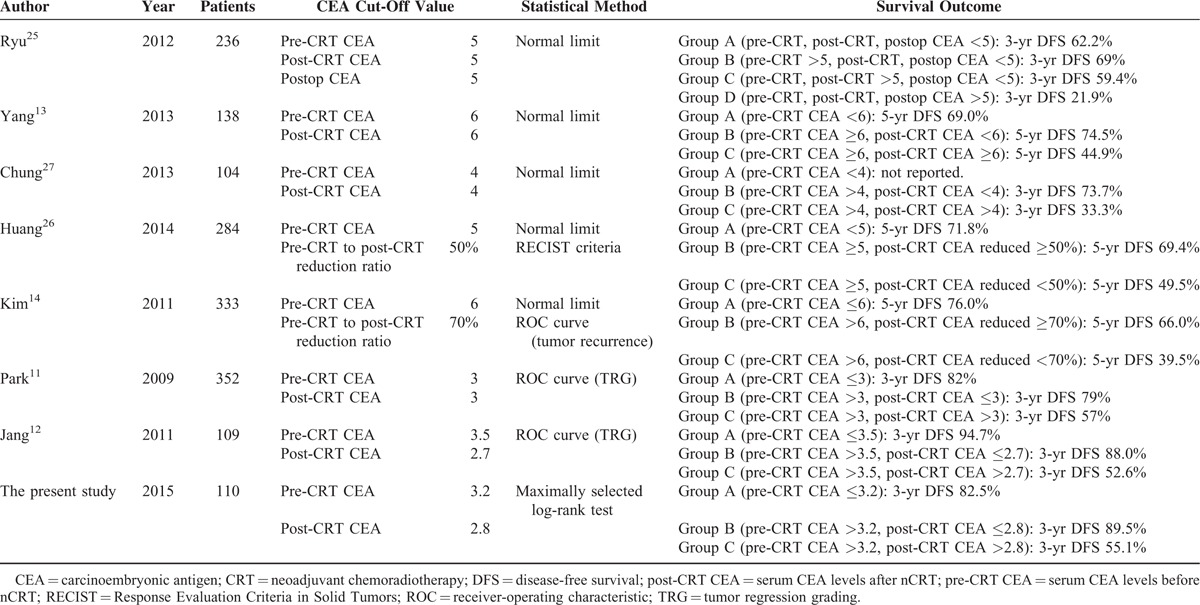
Summary of Reports Analyzing Association Between Carcinoembryonic Antigen Group and Prognosis

To predict prognosis according to CEA group, estimation of cut-off values of pre-CRT CEA and post-CRT CEA is the most important point. Comparing the studies listed in Table 4, several studies simply used a laboratory upper limit of normal in their institutions.^13,14,23–27^ In most cases, the value was 5 or 6 ng/mL. Although these levels have been widely used since the time CEA was adopted as a tumor marker in colorectal cancer, this usage was based on the reports investigating the prognostic value of the initial CEA level before the adoption of nCRT. A few studies determined cut-off values using receiver-operating characteristic (ROC) curves.^11,12,14^ In those studies, either tumor regression grade (TRG) or tumor recurrence was selected as an endpoint to generate the ROC curve. Only 1 study by Kim et al^14^ used tumor recurrence instead of TRG. Patients were then grouped according to the cut-off values calculated from the ROC curve. However, those cut-off values were actually optimal for predict of tumor regression or recurrence, not to directly predict the survival. In the current study, we chose a different statistical method to calculate an optimal cut-off value representing a direct association with DFS. The cut-off value was analyzed using a maximally selected log-rank test. Both DFS and the CEA level were used as variables in that statistical method. We calculated cut-off values that are associated with DFS directly, and this method might be more proper to identify the patients who are likely to show a good prognosis. Despite using a different statistical method, our cut-off values are consistent with previously reported values. Using elaborate analysis, we added weight to prognostic impact of CEA values.

In this study, DFS for distant metastasis was also analyzed. The 3-year DMFS rates were similar in Group A and Group B, but significantly lower in Group C (89.4% vs. 89.5% vs. 54.0%, respectively, *P* < 0.001). Of 23 patients who experienced distant metastasis, 18 patients (78.2%) were in Group C. The high distant metastasis rate in Group C is thought to contribute to the low 3-year DFS rate. Chung et al^27^ reported that the 3-year DMFS rate was 73.7% in the patients with preCRT CEA >4 ng/mL and post-CRT CEA ≤4 ng/mL, but 33.3% in the patients with post-CRT CEA >4 ng/mL (*P* = 0.018). Of 13 patients who demonstrated recurrence, 7 patients (53.8%) had metastasis to the liver and 4 patients (30.8%) had metastasis to the lung. Another report by Huang et al^26^ reported that liver metastasis occurred in 25.0% of patients with post-CRT CEA ≥5 ng/mL and this was a higher percentage than the 7.3% in patients with post-CRT CEA <5 ng/mL. The authors suggested that post-CRT CEA levels have a prognostic value for not only primary rectal cancer alone but also for liver metastasis. In this study, all patients were considered for adjuvant chemotherapy which is based on a 5-FU/leucovorin regimen, irrespective of serum CEA level. In some patients, recurrence was detected before completion of chemotherapy. For those patients, second-line chemotherapy was administered such as FOLFOX or FOLFIRI. Of the 41 patients in Group C, 8 patients (19.5%) showed recurrences before the completion of chemotherapy and were treated with an alteration of the chemotherapy regimen. This means that the patients in Group C have a possibility of occult metastases that were not detected on routine evaluation before treatment. After evaluating post-CRT CEA levels, more precise examinations may be considered to assess the presence of metastasis for patients in Group C. Liver MRI is a highly sensitive imaging modality to detect metastasis in the liver, in which is the most common metastatic site, and positron emission tomography-computed tomography (PET-CT) is another efficient modality providing systemic evaluation of metastasis.^28^ Although liver MRI and PET-CT are not routinely recommended to all patients under the current guidelines,^29^ patients in Group C are highly likely to benefit from these examinations.

The results of this study should be interpreted with caution because of the retrospective nature of this study and the relatively small number of patients. Although our results were obtained using rational and proper statistical analyses, they are not yet sufficient for generalization, and further large-scale validation studies are needed to confirm our results.

In conclusion, the post-CRT CEA level may be a useful prognostic factor for DFS and DMFS in patients whose prognosis cannot be predicted exactly by the pre-CRT CEA level alone in the neoadjuvant treatment era. CEA grouping that combines pre-CRT CEA and post-CRT CEA levels is an independent prognostic factor for DFS in locally advanced colorectal patients who received nCRT followed by surgical resection and would be helpful in the more accurate identification of patients at risk for poor prognosis.
